# Three Faces of Self-Destruction: A Case Series of Warm, Cold, and Mixed Autoimmune Hemolytic Anemia

**DOI:** 10.7759/cureus.109009

**Published:** 2026-05-17

**Authors:** Hema K, Rajashekar V, Mukil S, Mohammed Thaha S, Akshaya Kumar, Sahasyaa Adalarasan, TS Santhi

**Affiliations:** 1 Internal Medicine, Madras Medical College, Chennai, IND; 2 Medicine, Madras Medical College, Chennai, IND

**Keywords:** aiha, autoimmune hemolytic anemia, cold agglutinins, cold aiha, direct antiglobulin test, hemolysis, immunohematology, mixed aiha, warm aiha

## Abstract

Autoimmune hemolytic anemia (AIHA) is an immune-mediated disorder in which red blood cells are destroyed by autoantibodies. It is classified as warm, cold, or mixed depending on the type of antibody involved. The clinical presentation can range from mild anemia to severe hemolysis, and it may occur as a primary condition or secondary to infections, autoimmune disorders, or hematological malignancies. Diagnosis relies on clinical findings combined with immunohematological tests, specifically the direct antiglobulin test (DAT). We describe the cases of three patients with different types of AIHA.

The first patient was a 58-year-old woman who presented with severe anemia, low blood counts, and hepatosplenomegaly. Her blood tests revealed cold agglutinins and a positive DAT, and further evaluation identified B-cell acute lymphoblastic leukemia (B-ALL), confirming secondary cold AIHA. The second patient was a 55-year-old woman who presented with anemia, jaundice, and evidence of hemolysis, leading to a diagnosis of warm AIHA. The third patient was an 18-year-old woman who presented with severe anemia and splenomegaly. Her blood tests demonstrated both warm and cold antibody activity, and she was diagnosed with mixed AIHA. All three patients were treated with appropriate therapies and supportive care, resulting in clinical and hematological improvement.

This case series illustrates the different ways AIHA can present and highlights the need for detailed immunohematological testing to identify the exact type, as well as the differences in treatment and management across AIHA subtypes. It also emphasizes the importance of searching for an underlying cause, as this guides treatment and improves outcomes.

## Introduction

Autoimmune hemolytic anemia (AIHA) is an immune-mediated disorder in which red blood cells are destroyed by autoantibodies. It is classified into warm, cold, and mixed types according to the antibody involved and the temperature at which it reacts [1]. Warm AIHA is the most common form and is mediated by IgG antibodies [2], whereas cold AIHA is mediated by IgM antibodies that activate complement-mediated hemolysis [3]. Mixed AIHA exhibits features of both warm and cold antibody activity, making it a distinct but less common subtype [4]. The clinical presentation of AIHA is generally similar across all subtypes, with patients presenting with anemia, jaundice, and laboratory evidence of hemolysis. However, accurately identifying the subtype can be challenging, as routine investigations do not clearly differentiate between them [5].

The direct antiglobulin test (DAT) is the primary diagnostic tool, but further evaluation with monospecific testing and cold agglutinin studies is often required for accurate subtype classification. The DAT detects antibodies or complement components bound to the surface of red blood cells, confirming immune-mediated hemolysis. Monospecific testing determines whether the coating consists of IgG, the complement component C3d, or both, which helps differentiate warm and cold subtypes of AIHA. Cold agglutinin studies detect cold-reactive antibodies, usually IgM, that cause red blood cells to agglutinate at low temperatures, supporting the diagnosis of cold AIHA [6].

AIHA may occur as a primary condition or secondary to an underlying disorder, and identifying the clinical etiology is crucial in patient evaluation. Secondary causes commonly include autoimmune diseases, lymphoproliferative disorders, infections, and drug exposure [7]. Warm AIHA is more frequently associated with autoimmune conditions such as systemic lupus erythematosus (SLE) and with hematological malignancies, particularly lymphoproliferative disorders [2]. Cold AIHA is often linked to infections such as Mycoplasma pneumoniae and Epstein-Barr virus, as well as clonal B-cell disorders [8]. Drug-induced immune hemolytic anemia is another etiology, in which medications trigger antibody formation against red blood cells through immune-mediated mechanisms, with commonly implicated drugs including beta-lactam antibiotics and chemotherapeutic agents [9]. Mixed AIHA may be associated with autoimmune diseases, infections, or malignancies, but is often under-recognized due to overlapping clinical and serological features, potentially delaying diagnosis and management [10]. Distinguishing between warm, cold, and mixed AIHA is essential, as the underlying cause and management strategies differ between subtypes. 

This series emphasizes the importance of careful immunohematological evaluation and the need to identify both the subtype and any underlying cause for optimal management of AIHA. We report three patients, each representing a different type of AIHA. The first patient had cold AIHA secondary to B-cell acute lymphoblastic leukemia (B-ALL). The second patient had warm AIHA, and the third patient had mixed AIHA.

## Case presentation

Case 1: cold autoimmune hemolytic anemia

A 58-year-old female presented with complaints of easy fatigability and breathlessness on exertion for the past seven days. There was no other significant history. On clinical examination, the patient had pallor and icterus, as well as hepatosplenomegaly. There was no generalized lymphadenopathy. Laboratory investigations revealed severe anemia, with a hemoglobin level of 3.7 g/dL, leukopenia, with a total leukocyte count of 1.6 × 10³/µL, and thrombocytopenia, with a platelet count of 37 × 10³/µL, suggesting pancytopenia. Red cell indices at presentation showed a mean corpuscular volume (MCV) of 86.3 fL, mean corpuscular hemoglobin (MCH) of 26.7 pg, and mean corpuscular hemoglobin concentration (MCHC) of 31.0 g/dL. These findings were consistent with dimorphic anemia. Liver function tests showed hyperbilirubinemia, with a total bilirubin of 4.1 mg/dL, direct bilirubin of 1.6 mg/dL, and indirect bilirubin of 2.5 mg/dL, indicating hemolysis. The erythrocyte sedimentation rate was elevated (Table 1).

**Table 1:** Clinical presentation, hematological parameters, biochemical findings, immunohematological profile, underlying cause, and treatment details of the patient with cold AIHA Note: The initially low reticulocyte count (0.65%) was due to impaired erythropoietic response secondary to marrow infiltration and fibrosis associated with B-ALL, while the relatively low LDH level (150 U/L) reflected predominantly extravascular hemolysis and variability in the degree of ongoing red blood cell destruction AIHA: autoimmune hemolytic anemia; RBCs: red blood cells; IgM: immunoglobulin M; USG: ultrasonography; CT: computed tomography; LDH: lactate dehydrogenase; B-ALL: B-cell acute lymphoblastic leukemia; PRBC: packed red blood cells

Parameter	Findings	Reference value
Age in years/sex	58/female	—
Presentation	Easy fatigability, breathlessness on exertion (7 days)	—
Clinical examination	Pallor, icterus, hepatosplenomegaly; no lymphadenopathy	—
Hemoglobin	3.7 g/dL	12-16 g/dL
Total leukocyte count	1,600 cells/mm³	4,000-11,000 cells/mm³
Platelet count	37,000/mm³	1.5-4.5 × 10⁵/mm³
Peripheral smear	Dimorphic picture: microcytic hypochromic + normocytic normochromic RBCs with anisopoikilocytosis	Normal: normocytic normochromic RBCs
Reticulocyte count	0.65%	0.5-2.5%
Total bilirubin	4.1 mg/dL	0.3-1.2 mg/dL
Direct bilirubin	1.6 mg/dL	0-0.3 mg/dL
Indirect bilirubin	2.5 mg/dL	0.2-0.8 mg/dL
Lactate dehydrogenase	150 U/L	140-280 U/L
DAT (direct antiglobulin test)	Positive (C3d; IgM-mediated)	Negative
Other immunohematology	Cold agglutinins present	Absent
Anti-nuclear antibody (ANA)	Negative	Negative
Imaging/special tests	USG: hepatomegaly; CT chest: left lower lobe consolidation; bone marrow: B-ALL with fibrosis	—
Underlying cause	Secondary cold autoimmune hemolytic anemia associated with B-ALL	—
Treatment	Prednisolone 1 mg/kg/day + single-dose rituximab + 3 units PRBC transfusion + vitamin B12 (1000 mcg) + folic acid + B-complex + albendazole + supportive care + for underlying ALL induction therapy with asparaginase and vincristine was given. Following induction, maintenance therapy with methotrexate and 6-mercaptopurine was given	—

Further workup showed a positive DAT with cold agglutinins. DAT specificity showed C3d positivity, suggestive of cold antibody-mediated hemolysis. Screening for HIV and VDRL (Venereal Disease Research Laboratory) tests was negative. Renal function tests and serum electrolytes were within normal limits. Peripheral smear showed microcytic hypochromic anemia with anisopoikilocytosis, along with normocytic normochromic red blood cells, suggesting dimorphic anemia. Viral markers and ANA were negative. The thyroid profile was within normal limits, and the serum folate level was 7.25 ng/mL. Imaging showed hepatomegaly on ultrasonography, and CT of the chest revealed left lower lobe consolidation, suggesting active pulmonary infection.

In light of the pancytopenia and hepatosplenomegaly, a bone marrow evaluation was performed. Bone marrow biopsy showed increased blasts with grade 3 marrow fibrosis. Based on these findings, a diagnosis of B-ALL was established. Serum protein electrophoresis was normal, with no monoclonal band, ruling out monoclonal gammopathy-associated cold agglutinin disease. These findings supported a diagnosis of secondary cold AIHA associated with B-ALL.

The patient was treated with oral prednisolone at a dose of 1 mg/kg/day, along with a single dose of intravenous rituximab. In view of severe anemia, she received a transfusion of three units of packed red blood cells. Since the condition was secondary to underlying B-ALL, specific chemotherapy for ALL was also initiated. Induction therapy consisted of L-asparaginase and vincristine. Following induction, the patient was started on maintenance therapy with methotrexate and 6-mercaptopurine. Supportive treatment included injectable vitamin B12 (1000 mcg), oral folic acid, B-complex tablets, albendazole, and paracetamol.

On follow-up, hematological improvement was noted. Although the patient had evidence of immune-mediated hemolysis, the initial reticulocyte count was relatively low at 0.65%, likely due to impaired compensatory erythropoiesis secondary to bone marrow infiltration and grade 3 marrow fibrosis associated with the underlying B-ALL, as well as pancytopenia. The lactate dehydrogenase (LDH) level was also relatively low at 150 U/L, possibly reflecting predominantly extravascular hemolysis and variability in the degree and timing of ongoing red blood cell destruction. Following treatment with corticosteroids, rituximab, transfusion support, and chemotherapy directed toward the underlying B-ALL, the reticulocyte count increased to 4.59%, indicating recovery of marrow erythropoietic activity and improved compensatory response to anemia. Red cell indices also showed improvement, with MCV increasing from 86.3 fL to 91.9 fL and MCH increasing from 26.7 pg to 28.1 pg. At the same time, MCHC remained stable around 30-31 g/dL, reflecting stabilization of red blood cell morphology and improvement in erythropoiesis.

Case 2: warm autoimmune hemolytic anemia

A 55-year-old female presented with complaints of easy fatigability for 20 days, bilateral lower limb swelling for 20 days, and progressively worsening breathlessness for one week, corresponding to New York Heart Association class III. She also reported yellowish discoloration for one week and hematuria. There was no history of fever, giddiness, or palpitations. She was not a known case of hypertension, diabetes mellitus, chronic obstructive pulmonary disease, bronchial asthma, or epilepsy. On examination, the patient was conscious and oriented. Pallor and icterus were present, along with bilateral pedal edema. Oxygen saturation was normal on room air, and she was afebrile. Systemic examination was normal. Ultrasonography of the abdomen showed no significant abnormalities.

Laboratory investigations revealed severe anemia with a hemoglobin level of 5.6 g/dL, a total leukocyte count of 5.2 × 10³/µL, and a platelet count of 2.6 × 10⁵/µL. Red cell indices showed macrocytosis, with an MCV of 112 fL and MCH of 35.7 pg, along with an elevated red cell distribution width of 21%. Peripheral smear showed anisocytosis, macrocytes, schistocytes, elliptocytes, and pencil cells. Iron studies showed reduced serum iron (36 µg/dL), elevated total iron-binding capacity (352 µg/dL), and transferrin saturation of 23%. Vitamin B12 level was 214 pg/mL. The macrocytosis despite iron deficiency is explained by reticulocytosis, as reticulocytes are larger cells. A bone marrow biopsy was performed to rule out a secondary cause, and the findings were normal. Viral markers were negative (Table 2).

**Table 2:** Clinical presentation, hematological parameters, biochemical findings, immunohematological profile, underlying cause, and treatment details of the patient with warm AIHA AIHA: autoimmune hemolytic anemia; RBCs: red blood cells; IgG: immunoglobulin G

Parameter	Findings	Reference value
Age in years/sex	55/female	—
Presentation	Easy fatigability (20 days), bilateral pedal edema, breathlessness, jaundice, hematuria	—
Clinical examination	Pallor, icterus, and bilateral pedal edema	—
Hemoglobin	5.6 g/dL	12-16 g/dL
Total leukocyte count	5,200 cells/mm³	4,000-11,000 cells/mm³
Platelet count	2.6 lakh/mm³	1.5-4.5 × 10⁵/mm³
Peripheral smear	Macrocytes, anisocytosis, schistocytes, elliptocytes, pencil cells (dimorphic picture)	Normal: normocytic normochromic RBCs
Reticulocyte count	26.36%	0.5-2.5%
Total bilirubin	3.06 mg/dL	0.3-1.2 mg/dL
Direct bilirubin	0.82 mg/dL	0-0.3 mg/dL
Indirect bilirubin	2.24 mg/dL	0.2-0.8 mg/dL
Lactate dehydrogenase	381 U/L	140-280 U/L
DAT (direct antiglobulin test)	Positive (IgG)	Negative
Other immunohematology	No cold agglutinins detected	Absent
Anti-nuclear antibody (ANA)	Not done	Negative
Imaging/special tests	Ultrasonography abdomen: normal	—
Underlying cause	Primary warm autoimmune hemolytic anemia	—
Treatment	Prednisolone 1 mg/kg/day + folic acid + supportive care	—

Further evaluation revealed a reticulocyte count of 26.36% and an elevated LDH of 381 U/L, indicating ongoing hemolysis. Serum bilirubin was elevated, with a total bilirubin of 3.06 mg/dL and direct bilirubin of 0.82 mg/dL. Liver enzymes (aspartate aminotransferase (AST)/alanine aminotransferase (ALT)) were within normal limits. Renal function tests showed urea levels ranging from 26-33 mg/dL and creatinine levels between 0.6-0.8 mg/dL. Coagulation parameters, including prothrombin time (PT) (13.7 seconds) and international normalized ratio (INR) (1.17), were within normal limits. DAT was positive for IgG. Urine examination showed no significant abnormalities.

Based on the positive DAT, elevated reticulocyte count, raised LDH, and hyperbilirubinemia, the patient was diagnosed with warm AIHA. She was treated with oral prednisolone at a dose of 1 mg/kg/day, packed red blood cell transfusions, and folic acid supplementation. The patient showed clinical improvement with treatment, and her overall prognosis was favorable with appropriate follow-up and continued management.

Case 3: mixed autoimmune hemolytic anemia

**Figure 1:**
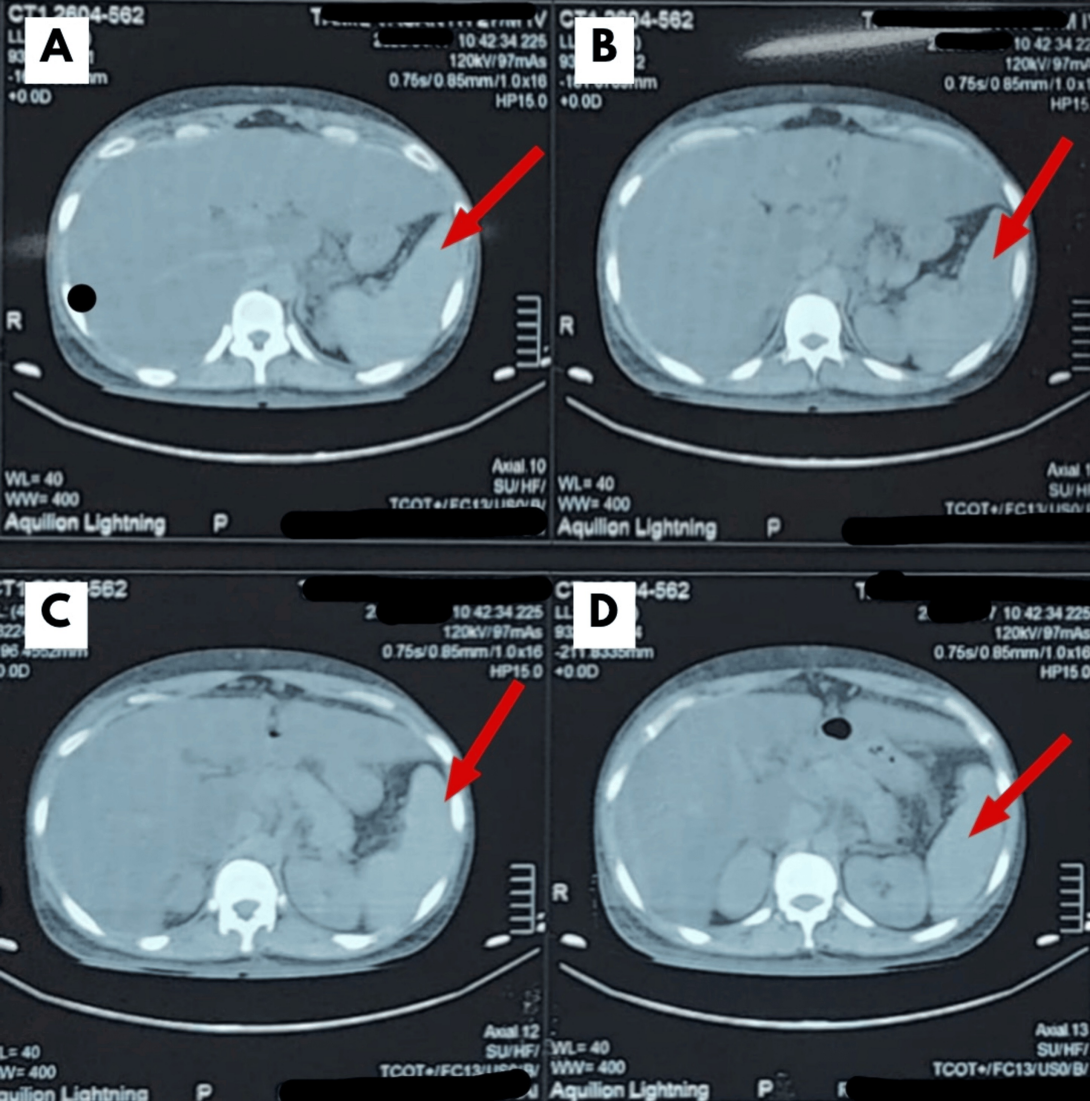
Axial contrast-enhanced CT scan of the abdomen showing an enlarged spleen (indicated by the red arrow) (A-D) Axial CT images at different levels demonstrate spleenomegaly, as indicated by red arrows. Splenomegaly in autoimmune hemolytic anemia occurs due to increased sequestration and extravascular destruction of antibody-coated red blood cells within the spleen, reflecting ongoing hemolysis and increased splenic activity CT: computed tomography

An 18-year-old female presented with complaints of breathlessness on exertion for 10 days and fever for the past two days. She also had a history of cough with yellow sputum for 10 days, which was not foul-smelling. Her menstrual cycles were regular, with menorrhagia occurring in each cycle. On general examination, the patient was conscious and oriented, with severe pallor. Her pulse rate was 112/min, suggesting tachycardia. Other vital signs were within normal limits. Abdominal examination revealed a soft abdomen with splenomegaly. Systemic examination was otherwise normal. Ultrasonography and CT of the abdomen showed mild splenomegaly (Figure 1). Splenomegaly in AIHA occurs due to increased sequestration and extravascular destruction of antibody-coated red blood cells within the spleen, reflecting ongoing hemolysis and increased splenic activity.

Laboratory investigations revealed severe hypochromic microcytic anemia with a hemoglobin level of 4.2 g/dL. Liver function tests showed a total bilirubin of 1.2 mg/dL, direct bilirubin of 0.4 mg/dL, and indirect bilirubin of 0.9 mg/dL. Red cell indices showed an MCV of 84 fL, MCH of 17 pg, and MCHC of 20 g/dL. Peripheral smear showed microcytic hypochromic red cells with anisopoikilocytosis, elliptocytes, tear drop cells, and polychromatophils. Reticulocyte count was 7%. Platelet count was 2.92 × 10⁵/µL. LDH was 352 IU/L. Renal function tests, electrolytes, thyroid profile, and urine analysis were within normal limits. An iron deficiency component was considered in light of the microcytic hypochromic anemia and history of menorrhagia (Table 3).

**Table 3:** Clinical presentation, hematological parameters, biochemical findings, immunohematological profile, underlying cause, and treatment details of the patient with mixed AIHA AIHA: autoimmune hemolytic anemia; RBCs: red blood cells; IgG: immunoglobulin G; IAT: indirect antiglobulin test; SSA: Sjögren's-syndrome-related antigen A; SLE: systemic lupus erythematosus

Parameter	Findings	Reference value
Age in years/sex	18/female	—
Presentation	Breathlessness (10 days), fever (2 days), cough with yellow sputum, menorrhagia	—
Clinical examination	Severe pallor, tachycardia, splenomegaly	—
Hemoglobin	4.2 g/dL	12-16 g/dL
Total leukocyte count	Within normal limits	4,000-11,000 cells/mm³
Platelet count	2.92 × 10⁵/mm³	1.5-4.5 × 10⁵/mm³
Peripheral smear	Microcytic hypochromic RBCs with anisopoikilocytosis, elliptocytes, tear drop cells, and polychromatophils	Normal: normocytic normochromic RBCs
Reticulocyte count	7 %	0.5-2.5%
Total bilirubin	1.2 mg/dL	0.3-1.2 mg/dL
Direct bilirubin	0.4 mg/dL	0-0.3 mg/dL
Indirect bilirubin	0.8 mg/dL	0.2-0.8 mg/dL
Lactate dehydrogenase	352 U/L	140-280 U/L
DAT (direct antiglobulin test)	Positive (IgG + C3d)	Negative
Other immunohematology	Panagglutination, positive IAT, cold antibody titre 1:128, autocontrol positive at 4°C, 22°C, 37°C	Absent
Anti-nuclear antibody (ANA)	Positive (3+), anti-SSA 3+	Negative
Imaging/special tests	Ultrasonography: mild splenomegaly	—
Underlying cause	Mixed autoimmune hemolytic anemia secondary to underlying autoimmune disease (SLE/Sjögren’s overlap suspected)	—
Treatment	Prednisolone 100 mg/day (5 days) tapered to 40 mg/day + azithromycin 500 mg/day + IV iron sucrose 200 mg + TMP-SMX 480 mg (twice weekly) + ranitidine + calcium + oral iron	—

Immunohematological evaluation confirmed the diagnosis. Blood grouping showed O RhD-positive status. DAT was positive, with monospecific testing showing both IgG and C3d positivity. Indirect antiglobulin test was positive, and antibody screening with eluate showed panagglutination. Autocontrol was positive at 4°C, 22°C, and 37°C, with a cold antibody titer of 1:128. Further evaluation showed a positive antinuclear antibody (ANA) with a cytoplasmic pattern (3+ intensity) and strongly positive anti-SSA (3+), suggesting an underlying autoimmune etiology like SLE with Sjögren’s overlap. Broad thermal amplitude testing demonstrated antibody reactivity across multiple temperatures, supporting the coexistence of warm and cold autoantibody activity and confirming the diagnosis of mixed AIHA. Based on these findings, the patient was diagnosed with mixed AIHA secondary to an underlying autoimmune disease.

The patient was treated with oral prednisolone at an initial dose of 100 mg/day, given as 50 mg in the morning, 25 mg in the afternoon, and 25 mg in the evening, for the first five days. This was subsequently tapered to 40 mg/day in divided doses of 20 mg in the morning and 20 mg in the afternoon. She also received azithromycin 500 mg once daily for upper respiratory tract infection, ranitidine 150 mg, and intravenous iron sucrose 200 mg diluted in normal saline. Trimethoprim-sulfamethoxazole (480 mg) was administered twice weekly as prophylaxis. During hospitalization, she showed hematological improvement, with hemoglobin rising to 6.3 g/dL, MCV to 82.4 fL, MCH to 21 pg, MCHC to 25.6 g/dL, and platelet count to 3.65 × 10⁵/µL after four days of therapy.

The patient improved clinically and hematologically during the hospital stay. At discharge, she was conscious, afebrile, hemodynamically stable, non-dyspneic, and adequately hydrated. She was advised to take oral prednisolone at a dose of 1 mg/kg/day and hydroxychloroquine 200 mg once daily at night. She was also prescribed oral iron supplementation, along with ranitidine and calcium. She was advised to follow an iron-rich diet and regular follow-up for monitoring of hemoglobin levels and disease activity.

## Discussion

AIHA is an immune-mediated disorder characterized by the premature destruction of red blood cells [5]. It is classified into warm, cold, and mixed subtypes based on the type of autoantibody and its thermal reactivity, which affects diagnosis and management, because each subtype differs in pathophysiology, site of hemolysis, associated conditions, and response to therapy [6]. Warm AIHA is mediated by IgG antibodies and results in extravascular hemolysis, predominantly in the spleen. Cold AIHA is mediated by IgM antibodies that activate complement, leading to hemolysis that may be intravascular or extravascular. Mixed AIHA involves the coexistence of warm autoantibodies with complement-mediated hemolysis, producing complex serological findings [6]. Complement-mediated red cell destruction plays an important role, particularly in cold and mixed AIHA [7].

Diagnosis requires the integration of clinical and laboratory findings. The direct antiglobulin test is the primary diagnostic tool for AIHA. Additional laboratory findings include anemia, reticulocytosis, elevated lactate dehydrogenase, indirect hyperbilirubinemia, and reduced haptoglobin [5]. Cold agglutinin titers and thermal amplitude testing are useful for suspected cold and mixed AIHA, while monospecific DAT can help differentiate between IgG- and complement-mediated hemolysis [6].

Cold AIHA is associated with lymphoproliferative disorders and typically requires evaluation for an underlying disease [8]. Drug-induced immune hemolysis is another secondary cause that should also be considered [9]. Mixed AIHA is less common and may be under-recognized due to overlapping serological features. It is characterized by the presence of both IgG antibodies and complement-mediated hemolysis [10]. A systematic review has demonstrated that mixed AIHA accounts for a small proportion of cases and is associated with autoimmune conditions and hematological malignancies [11]. Previous studies have reported mixed AIHA presenting with severe hemolysis and diagnostic challenges. Patients often demonstrate both IgG positivity and clinically significant cold agglutinins, which contribute to increased hemolysis [12]. Serological studies have shown panagglutination and broad thermal amplitude, factors that can complicate the interpretation of immunohematological testing [13].

In the present series, similar patterns were observed across the three cases. The cold AIHA case demonstrated C3d positivity with cold agglutinins and was secondary to an underlying hematological malignancy (B-cell acute lymphoblastic leukemia), consistent with the established association of cold AIHA with lymphoproliferative disorders [8]. The warm AIHA case showed IgG positivity on DAT with features of extravascular hemolysis and responded to corticosteroid therapy, consistent with previous reports [6].

The mixed AIHA case in this series showed both IgG and C3d positivity on monospecific DAT, along with panagglutination and a significant cold agglutinin titer, reflecting the dual pathophysiology described in prior studies [12-13]. The presence of a broad thermal amplitude and a positive autocontrol at multiple temperatures further supports the coexistence of warm and cold antibody activity. In addition, the association with a positive antinuclear antibody suggests an autoimmune background, as reported in mixed AIHA [11]. Compared to previously reported cases, the mixed AIHA patient in this series also presented with features indicative of an infectious trigger, which may have contributed to disease onset. The coexistence of iron deficiency added additional complexity to the clinical picture, highlighting the diagnostic challenges in such cases.

Management of AIHA varies according to subtype and severity. In warm AIHA, corticosteroids are the first-line treatment and are initiated as prednisolone at a dose of 1 mg/kg/day [6]. Rituximab is used in steroid-refractory cases [14]. In cold AIHA, corticosteroids have limited efficacy, and management includes avoidance of cold exposure and treatment of the underlying disorder, with rituximab-based therapy reserved for symptomatic patients [8]. Mixed AIHA requires an individualized approach due to its dual pathophysiology [10], where corticosteroids are initiated, but additional therapy, such as rituximab, may be necessary for sustained remission [11].

In the present series, the warm AIHA patient showed improvement with corticosteroid therapy alone. The cold AIHA patient required a multimodal approach, including corticosteroids, rituximab, transfusion support, and chemotherapy targeting the underlying B-ALL. The mixed AIHA patient showed hematological improvement following corticosteroid therapy combined with supportive management, including iron supplementation and treatment of intercurrent infection (Table 4). In the present cold AIHA case, the reticulocyte count was initially low despite severe anemia and evidence of hemolysis. This finding was likely due to impaired compensatory erythropoiesis caused by bone marrow infiltration and grade 3 marrow fibrosis associated with the underlying B-ALL. The relatively low LDH level (150 U/L) reflected predominantly extravascular hemolysis and variability in the degree of ongoing red blood cell destruction.

**Table 4:** Summary of clinical presentation, examination findings, laboratory parameters including immunohematological profile, and treatment in patients with cold, warm, and mixed AIHA AIHA: autoimmune hemolytic anemia; RBCs: red blood cells; IgM: immunoglobulin M; IgG: immunoglobulin G; IAT: indirect antiglobulin test; SSA: Sjögren's-syndrome-related antigen A; USG: ultrasonography; CT: computed tomography; B-ALL: B-cell acute lymphoblastic leukemia; SLE: systemic lupus erythematosus; PRBCs: packed red blood cells

Parameter	Case 1: cold AIHA	Case 2: warm AIHA	Case 3: mixed AIHA
Age in years/sex	58/female	55/female	18/female
Presentation	Easy fatigability, breathlessness on exertion (7 days)	Easy fatigability (20 days), bilateral pedal edema, breathlessness, jaundice, hematuria	Breathlessness (10 days), fever (2 days), cough with yellow sputum, menorrhagia
Clinical examination	Pallor, icterus, hepatosplenomegaly; no lymphadenopathy	Pallor, icterus, and bilateral pedal edema	Severe pallor, tachycardia, splenomegaly
Hemoglobin (12-16 g/dL)	3.7 g/dL	5.6 g/dL	4.2 g/dL
Total leukocyte count (4,000-11,000 cells/mm³)	1,600 cells/mm³	5,200 cells/mm³	Within normal limits
Platelet count (1.5-4.5 × 10⁵/mm³)	37,000/mm³	2.6 lakh/mm³	2.92 lakh/mm³
Peripheral smear	Dimorphic picture: microcytic hypochromic + normocytic normochromic RBCs with anisopoikilocytosis	Macrocytes, anisocytosis, schistocytes, elliptocytes, pencil cells (dimorphic picture)	Microcytic hypochromic RBCs with anisopoikilocytosis, elliptocytes, tear drop cells, and polychromatophils
Reticulocyte count (0.5-2.5%)	0.65%	26.36%	7%
Total bilirubin (0.3-1.2 mg/dL)	4.1 mg/dL	3.06 mg/dL	1.2 mg/dL
Direct bilirubin (0-0.3 mg/dL)	1.6 mg/dL	0.82 mg/dL	0.4 mg/dL
Indirect bilirubin (0.2-0.8 mg/dL)	2.5 mg/dL	2.24 mg/dL	0.8 mg/dL
Lactate dehydrogenase (140-280 U/L)	150 U/L	381 U/L	352 U/L
DAT (direct antiglobulin test)	Positive (C3d; IgM-mediated)	Positive (IgG)	Positive (IgG + C3d)
Other immunohematology	Cold agglutinins present	No cold agglutinins detected	Panagglutination, positive IAT, cold antibody titre 1:128, autocontrol positive at 4°C, 22°C, 37°C
Anti-nuclear antibody (ANA)	Negative	Not done	Positive (3+), anti-SSA 3+
Imaging/special tests	USG: hepatomegaly; CT chest: left lower lobe consolidation; bone marrow: B-ALL with fibrosis	Ultrasonography abdomen: normal	Ultrasonography: mild splenomegaly
Underlying cause	Secondary cold autoimmune hemolytic anemia associated with B-ALL	Primary warm AIHA	Mixed autoimmune hemolytic anemia secondary to underlying autoimmune disease (SLE/Sjögren’s overlap suspected)
Treatment (present cases)	Prednisolone 1 mg/kg/day + single-dose rituximab + 3 units PRBC transfusion + vitamin B12 (1000 mcg) + folic acid + B-complex + albendazole + supportive care + for underlying ALL induction therapy with asparaginase and vincristine was given. Following induction, maintenance therapy with methotrexate and 6-mercaptopurine was given	Prednisolone 1 mg/kg/day + folic acid + supportive care	Prednisolone 100 mg/day (5 days) tapered to 40 mg/day + azithromycin 500 mg/day + IV iron sucrose 200 mg + TMP-SMX 480 mg (twice weekly) + ranitidine + calcium + oral iron
First-line treatment (literature)	Avoid cold exposure, supportive care	Prednisolone 1 mg/kg/day	Prednisolone 1 mg/kg/day
Second-line/specific therapy (literature)	Rituximab 375 mg/m² weekly × 4 weeks; sutimlimab (fixed IV dosing)	Rituximab 375 mg/m² weekly × 4 weeks	Rituximab ± immunosuppressants
Key points (literature)	Steroids less effective	Steroid responsive; most common	Often needs combination therapy

The three cases demonstrated the clinical and immunohematological variability of autoimmune hemolytic anemia and highlighted the importance of subtype-specific evaluation to guide appropriate management. Although all patients presented with features of hemolysis, such as anemia, elevated bilirubin, raised lactate dehydrogenase, and reticulocytosis, the serological profiles varied significantly between subtypes. Warm AIHA showed IgG positivity on monospecific DAT and responded well to corticosteroid therapy. Corticosteroids are effective in warm AIHA because they suppress autoantibody production and reduce splenic clearance of antibody-coated red blood cells. In contrast, cold AIHA demonstrated complement-mediated hemolysis with C3d positivity and cold agglutinin reactivity, reflecting IgM-mediated red cell agglutination and complement activation at lower temperatures.

Since hemolysis in cold AIHA is primarily complement-mediated, corticosteroids are often less effective, and management focuses on avoidance of cold exposure, supportive care, and treatment of the underlying secondary condition. In the present case, cold AIHA was secondary to B-cell acute lymphoblastic leukemia, and chemotherapy targeting the underlying malignancy played an important role in controlling hemolysis. The mixed AIHA case showed overlapping features of both warm and cold antibody-mediated hemolysis, resulting in greater diagnostic complexity and a variable therapeutic response. Such cases may require a combination of approaches, including corticosteroids, immunosuppressive therapy, transfusion support, and close monitoring due to the coexistence of multiple immune mechanisms. Thus, differences in treatment response among the three cases reflected distinct underlying pathophysiological mechanisms and associated secondary conditions, emphasizing the importance of accurate serological classification for individualized management and prognostic assessment.

Taken together, this case series highlights the varied presentations of AIHA and emphasizes the importance of careful immunohematological evaluation. Early identification of the subtype and any associated conditions is crucial for guiding treatment and improving outcomes. This series contributes to the existing literature by demonstrating the coexistence of all three major AIHA subtypes within a single clinical setting.

## Conclusions

AIHA is an immune-mediated condition in which red blood cells are destroyed by autoantibodies and is classified as warm, cold, or mixed type. It can present with varying severity and may occur as a primary condition or secondary to infections, autoimmune disorders, or hematological malignancies, with diagnosis based on clinical findings and immunohematological tests, especially the DAT. In this case series, we describe three patients with different types of AIHA: a 58-year-old woman with cold AIHA secondary to B-ALL, a 55-year-old woman with warm AIHA, and an 18-year-old woman with mixed AIHA showing both warm and cold antibody activity. Although all three patients presented with features of hemolysis, the serological findings, underlying associations, and treatment approaches varied according to subtype and the immune mechanisms involved. Warm AIHA showed IgG-mediated extravascular hemolysis with a good response to corticosteroid therapy, while cold AIHA demonstrated complement-mediated hemolysis, requiring evaluation for secondary causes and therapy directed at the underlying malignancy. The mixed AIHA case exhibited overlapping warm and cold antibody activity, resulting in increased diagnostic complexity and necessitating individualized management. All patients showed clinical and hematological improvement with appropriate, subtype-specific therapy.
